# Metagenomic analysis of UK retail foods finds limited evidence for associations between food production method and antimicrobial resistance gene burden

**DOI:** 10.1099/mgen.0.001705

**Published:** 2026-04-29

**Authors:** Sam A. Mellor, Samuel J. Bloomfield, Raphaëlle Palau, George M. Savva, John Wain, Alison E. Mather

**Affiliations:** 1 Quadram Institute Bioscience, Norwich Research Park, Norwich, NR4 7UQ, UK; 2 Centre for Microbial Interactions, Norwich Research Park, Norwich, NR4 7UG, UK; 3 University of East Anglia, Norwich Research Park, Norwich, NR4 7TJ, UK

**Keywords:** antimicrobial resistance, food microbiome, food safety, metagenomics

## Abstract

Food is produced by a range of methods including extensive (organic and free range), intensive (conventional) and wild-caught production systems. Antimicrobial use varies between different food production systems, which may affect the microbial populations as well as the prevalence and diversity of antimicrobial resistance genes (ARGs) found on food at retail. In this study, shotgun metagenomics was used to investigate the microbial and ARG composition of 25 pork, 33 beef, 33 lamb, 60 chicken, 31 salmon and 41 leafy green samples collected in Norfolk, England, and labelled as extensive, wild caught or intensive. Food microbiomes consisted predominantly of spoilage-associated organisms including *Pseudomonas*, *Lactococcus* and *Psychrobacter*. Significant differences in bacterial diversity were found between intensive and extensive systems on chicken, and 22 differentially abundant genera were identified between production systems across beef, chicken and salmon. Genes conferring resistance to tetracyclines and beta-lactams comprised the majority of the food resistome across all commodities. Across most measures used to compare food resistomes between production methods, no significant differences were detected, except on chicken and salmon where differences in beta-diversity between production methods were detected, albeit with low effect sizes. Overall, these results suggest that differently produced foods, at least when tested at retail and in this region, may present a similar risk of antimicrobial resistance across the commodities investigated within this study. However, specific associations were identified with the microbial composition across chicken, beef and salmon, suggesting that production method may drive some variation in the microbial population structure on food products. Additional work at the farm or food processing levels is required to identify the drivers of these differences between production systems.

**Impact Statement**

Organic and free-range (here, collectively termed ‘extensive’) and wild-caught food production systems employ differing animal husbandry and antimicrobial stewardship practices to conventional (‘intensive’) production. These varying practices may impact the microbial contamination and antimicrobial resistance (AMR) burden on foods available to consumers. This study represents the largest effort to date applying shotgun metagenomics as an organism-agnostic approach to investigate whether or not these differences in farming and production practices translate to differences in the microbiome and resistome of retail foods. We investigate the microbial and AMR gene content of six food commodities, characterising variations in diversity and abundance between retail products produced via different systems. Our analysis finds minimal association of food production method with the foodborne AMR gene content of the UK retail foods studied. While antimicrobial use is a known driver of AMR, our results show that by the time foods produced by these different systems reach the level of the consumer, there are no detectable differences in AMR gene burden, at least in this setting. More work is needed to understand if these results are consistent with foods produced in other regions, which may be under different selection pressures.

## Data Summary

The authors confirm that all supporting data, code and protocols have been provided within the article or through supplementary data files. The datasets supporting the conclusions of this article are available in the European Nucleotide Archive (ENA) and Sequence Read Archive (SRA) repository, under BioProject accession Nos. PRJEB104242 and PRJNA849983, respectively. Accessions for individual samples are given in Table S1. All code used to perform analyses in this study is available on Zenodo (DOI: 10.5281/zenodo.17279294).

## Introduction

Food is produced by different methods including organic and free-range (here, collectively termed ‘extensive’) and conventional (intensive) methods which use different animal husbandry and antimicrobial stewardship practices [1]. Organic food sales are increasing worldwide due to perceived health benefits for consumers and a broadening awareness of animal welfare and sustainability of production [2,3].

Foodborne disease is an important health issue, with ~2.4 million cases in the UK annually [4]. This is compounded by antimicrobial resistance (AMR), as the treatment of drug-resistant infections may fail [5]. Antimicrobial usage is often associated with the prevalence of AMR organisms [6,7], and in recent years, there have been concerted efforts in many parts of the world to reduce antimicrobial use in food-producing animals. However, different farming practices may influence the risk of foodborne disease through differential selection for AMR. For example, UK organic farming regulations prohibit antimicrobial metaphylaxis, only permitting antimicrobial use in individual animals [1]. Both organic and free-range animals have increased outdoor access, are kept at decreased stocking density and are typically slaughtered at older ages compared to intensively farmed livestock [1,8, 9]. These differences may influence the AMR gene (ARG) content of foods at retail.

Metagenomics provides insight into the composition of ARGs across a microbial community without restriction to specific (groups of) organisms. Previous metagenomic studies have found higher abundance of ARGs, including tetracycline, aminoglycoside and macrolide-lincosamide-streptogramin resistance mechanisms from conventionally produced livestock compared to other production systems [10,11]. However, others have found no significant ARG differences [12]. Furthermore, the abundance of ARGs may decrease during food processing such that variation between production systems at slaughter/harvest may not be maintained at retail [13,16].

Few metagenomic studies have investigated the distribution of ARGs on foods from different production methods at retail. A Brazilian study reported significant increases in the total abundance of ARGs and mobile genetic elements in conventionally produced frozen chicken compared to organic and antibiotic-free samples [17]. However, the samples in this study were pooled together, limiting the insights available on individual samples. A previous UK study suggested there may be an increase in ARG concentration on aquacultured salmon products compared to wild caught [18]. Conversely, a US study investigating longitudinal effects in retail chicken breast microbiomes from four processing facilities found farm-level antibiotic usage claims had no effect on the microbiome composition [19]. Variation in previous findings may stem from differences in production practices between commodities and jurisdictions. This highlights the need for studies examining a broad range of different food commodities to build a comprehensive picture of the effect of production method on foodborne AMR.

Given the complexity of food systems, investigations from different geographic regions spanning multiple food commodities are needed to inform the food production industry and policy makers on the potential risks of AMR associated with different production systems. This study aims to address these gaps, investigating differences in the microbiome and resistome (total population of ARGs in an environment) of intensively and extensively produced UK retail products across a range of food commodities.

## Methods

### Sample collection and processing

Food samples were collected across three studies. Between February 2022 and May 2024, 167 retail food samples were collected from retail stores in and around Norwich, UK. These were combined with 91 samples from Bloomfield *et al*. collected from May 2018 to November 2019 from across Norfolk, UK, from which metagenomic DNA had been extracted, to ensure approximately equal numbers of samples from extensive and intensive production methods for each commodity [18]. Commodities included within the study were selected based on differences in legislation between production methods that may influence the risk of AMR, or those that may be consumed raw such that the risk of AMR may be elevated. Food commodities included raw beef, lamb, chicken, pork and salmon and pre-packaged leafy greens. Food samples included a wide range of products including different suppliers and meat cuts or leaf species.

Sample collection was carried out following the protocol of Janecko, Zamudio *et al*. [20]. Briefly, within date (use-by for meats, best-before for leafy greens), food samples were purchased and transported to the Quadram Institute Bioscience laboratory, Norwich, UK, with a cold chain maintained between 2 °C and 8 °C. Upon arrival, samples were stored in the laboratory cold room at 4 °C and processed within 72 h. Packaging was wiped with disinfectant to prevent cross-contamination. For all samples collected before September 2023, 100 g portions of the samples were transferred using sterilised equipment into stomacher bags (Corning, New York, USA), and 225 ml buffered peptone water (BPW) [Southern Group Laboratory (SGL), Corby, UK] was added. For samples collected after this date, the method was adapted to yield a higher concentration of metagenomic DNA post-extraction. For these samples, portions between roughly 100 and 200 g were weighed, and their mass was recorded. These were added to stomacher bags with 100 ml of BPW. To monitor potential reagent contamination, a blank was run in parallel for each sampling trip consisting of 225 ml (100 ml post September 2023) of BPW. Samples were then stomached at 100 r.p.m. for 30 s (Seward stomacher 400C laboratory blender, Worthing, UK). Where samples contained bones or hard fat, the stomacher bag was massaged by hand for 2 min instead. From the filtered side of the bag, 10 ml of BPW was taken for metagenomic DNA extraction.

### Host DNA depletion and metagenomic DNA extraction

One of the challenges in food metagenomics is the high proportion of host DNA recovered compared to bacterial DNA [14]. To combat this, host DNA depletion was performed for samples collected as described by Bloomfield *et al*. utilising a saponin-based differential lysis approach, limiting the recovery of host DNA and allowing greater resolution of the bacterial DNA [18]. DNA was extracted using a Maxwell RSC PureFood Pathogen Kit (Promega, Madison, Wisconsin, USA) on a Maxwell RSC 48 automated extraction system according to the manufacturer’s instructions. A Qubit dsDNA HS assay kit (ThermoFisher Diagnostics, Rochford, UK) was used to measure DNA concentration post-extraction. Blank samples had undetectable DNA concentrations and so were spiked with phiX174 RF1 DNA (New England Biolabs, Ipswich, MA, USA) to a concentration of 0.5 ng µl^−1^ for sequencing.

### Quantitative PCR

Sample bacterial DNA was measured by quantitative PCR (qPCR) post-host DNA depletion. Bacterial DNA concentration was quantified by the TaqMan method targeting the 16S rRNA gene [primers (forward/reverse): BactQuant-F/BactQuant-R. Probe: BactQuant-P] [21] using the PrecisionFAST qPCR Master Mix (PrimerDesign, Southampton, UK). Probes and primers were synthesised by Integrated DNA Technologies UK, Ltd.

### Illumina sequencing

Nextera DNA flex library prep kits (Illumina, San Diego, CA, USA) were used to prepare libraries from the extracted metagenomic DNA. Libraries for 15 samples were pooled and sequenced on an Illumina NovaSeq, while a pool of the remaining libraries was sequenced on an Illumina NextSeq. All samples were sequenced as 150 bp paired-end reads with a target depth of 8 Gb per metagenome. Samples for which insufficient DNA concentration (less than 5 ng µl^−1^) was recovered or inadequate sequencing depth (less than one million reads) was achieved were removed from the analysis.

### Read quality control and taxonomic classification

Reads from negative controls (blanks) were aligned to the PhiX174 genome using BBsplit (BBmap suite, 39.01) [22], and mapped reads were removed from the analysis. For all sequences, DNA reads were trimmed with FastP 0.23.1 [23] using the default parameters, and fastQC 0.11.8 [24] and MultiQC 1.7 [25] were used to assess the quality of raw/trimmed reads. Trimmed reads were classified using MetaPhlAn 4.0.3 [26] against the CHOCOPhlAn database (version January 2021) with the additional parameters -t rel_ab_w_read_stats (output relative abundance with read numbers) and --unclassified_estimation (output unclassified estimations). The results files were merged to form a single feature table consisting of the number of reads assigned to each taxon in each sample. For differential abundance analysis of bacteria between production methods, genus-level classifications were retrieved from each MetaPhlAn4 results file and combined into a feature table.

### Resistance gene identification and abundance calculation

ARGs and plasmid replicons were identified within metagenomes using KMA 1.4.9 [27] to align trimmed reads to the ResFinder [28] and PlasmidFinder [29] databases, respectively. KMA was run with the following flags: -ef (output extra features including mapping statistics), -1 t1 (force reads to map one to one with templates in the database), -apm f (force reads to pair) and -mem-mode (more memory efficient). Sample results were compiled into a single file and ARGs/replicons below 90% query identity and 60% template coverage were filtered out. The relative abundance of ARGs identified by KMA was calculated as fragments per kilobase per million mapped reads by taking the reads mapped to ARGs, multiplied by 10^9^ (scaling factor), divided by the product of the template length and the total number of reads [30,31].

Hereafter, unique ARGs are defined as those hits (reads mapped to a template in the ResFinder database by KMA) corresponding to a unique gene ID within the database. When reads mapped to multiple templates of the same gene ID, the calculated abundance of these was combined, and they were counted as one unique ARG for the analysis of diversity.

### Resistance gene concentration calculations

The concentration of bacteria and ARGs on food samples was calculated from qPCR and metagenomic data following the method described in Bloomfield *et al*. [18] with the following modifications: For samples collected before September 2023, food mass was estimated at 100 g, and the volume of BPW used was 0.225 l. Samples collected since then were weighed and 0.1 l BPW per sample was used.

### Statistical analysis

Analysis of taxonomic and ARG diversity was conducted in R 4.3.2 [32] using the phyloseq 1.42.0 [33] and vegan 2.6.4 [34] packages for phylogenomics and diversity analysis. Taxonomic data were agglomerated at the genus level, and samples with less than one million reads were removed. Taxonomic and ARG read counts were rarefied to the depth of the lowest sample (1,295,949 reads) using vegan. Within-sample (alpha) diversity was calculated with vegan using richness, Shannon index [35], and inverse Simpson’s index [36] measures. Richness measures were log transformed prior to statistical analysis, and a pseudo-count of one was applied to the single sample with no reads aligning to ARGs. To assess the impact of the choice of pseudo-count, three further values were tested: two, 10 and 0.5, and the models produced were compared. In all cases, the choice of pseudo-count did not have an effect on the model outcome. Due to the similarities between organic and free-range production methods, these were grouped into one ‘extensive’ category.

For the analysis of both the microbiota and resistome, linear models (LMs) were used to examine the association between production method and each of Shannon index, richness and inverse Simpson’s index. Covariates potentially influencing the microbiota and resistome on foodstuffs to be included in the models were identified from the previous literature [19,20, 37]. The final list of covariates included calendar date [encoded in quarter years (season)] as a fixed effect for all commodities except chicken (where convenience sampling precluded its inclusion), sample cut as a fixed effect for all meat commodities, main component vegetable as a fixed effect for leafy greens and processing facility (proxied with supplier code) as a random effect for beef, lamb, pork and leafy greens.

Separate models were estimated for each commodity, due to the inherent differences in production between different commodities. For models including random effects, lmerTest 3.1–3 was used to construct linear mixed effects models (LMEs) [38,39]. As well as the covariates outlined above, the log-transformed total read count was included as a fixed effect to account for variability in sequencing depth between samples. Model assumptions were visually checked using the performance package [40]. Post hoc pairwise comparison of production methods was conducted using emmeans [41]. Alpha diversity was visualised with ggplot2 3.4.4 [42], and gtsummary 2.0.0 was used to produce tables of the regression coefficients [43].

The differential abundance of taxa and ARGs between production methods for each commodity under study was investigated using the ALDEx2 1.34 R package [44]. For the analysis of taxa, the genus-level feature table was used as input. For the analysis of ARGs, the KMA results were converted into a feature table consisting of the number of reads mapped to each ARG identified (columns) in each sample (rows). The resulting feature tables were split by commodity and input to ALDEx2 with metadata to test for associations between genera or ARGs and production methods. ALDEx2 fit a generalised LM to the centred log-ratio transformed read counts and calculated the effect as the median difference between groups divided by the median difference within groups, using intensive production as the reference group. Models were constructed with the production method as the covariate of interest. To assess associations between unknown or wild-caught and extensive samples, a second ALDEx2 model was fit with ‘extensive’ as the reference group. The *P*-values were corrected for multiple hypothesis testing across all models generated via the Benjamini–Hochberg approach. Results were reported as associated with a production method if they had a corrected *P*<0.05 and an effect size >1 (suggesting the between-group differences were larger than the within-group dispersion).

To investigate differences in the contamination of genera containing known foodborne pathogens between production systems, Fisher’s Exact tests were used to examine the presence/absence of each pathogen identified from the rarefied taxonomic data between production methods for each commodity. *Campylobacter*, *Salmonella*, *Listeria*, *Vibrio*, *Escherichia* and *Yersinia* were selected as genera containing well-described foodborne pathogens, including key pathogens monitored by the UK Food Standards Agency [45]. For each commodity, model results from each pathogen were corrected for multiple comparisons using Bonferroni correction.

Between-sample (beta) diversity was measured using Bray–Curtis dissimilarity [46]. Samples with less than one million reads were excluded, and the dataset was relativised before calculating dissimilarity scores. The dissimilarity matrix was visualised using non-metric multidimensional scaling, and samples were coloured by production method. Samples where no ARGs were identified were filtered out of the dataset prior to analysis. Adonis2 [34] was used to perform permutational multivariate ANOVA (PERMANOVA) on the dissimilarity matrix to investigate the association of production method with Bray–Curtis dissimilarity. Covariates outlined above, and log-transformed total read count, were included in the models as fixed effects. Effect size was calculated as the distance between group centroids (partial omega squared) using the MicEco (0.0.19) package [47]. Production method dispersions were calculated from Bray–Curtis dissimilarities using the betadisper function from vegan, and variability between groups was compared using the permutest function [34]. Bonferroni correction was used to adjust the *P*-values from adonis2 and permutest models across the commodities to correct for multiple comparisons.

The association of production method with the unique number of plasmid replicons and ARG and bacterial concentration was assessed using LMs/LMEs. Plasmid replicons were rarefied in line with the taxonomic and ARG diversity estimates. Bacterial concentrations and plasmid replicon counts were log transformed, and covariates were included in the analysis as outlined above. As previously, a pseudo-count of one was applied to samples containing no reads aligning to ARGs or replicons. Post hoc comparisons were performed using emmeans [41], and Bonferroni correction was used to correct for multiple comparisons.

## Results

Metagenomic DNA was isolated from a total of 258 food samples purchased from retailers in and around Norwich, Norfolk, UK, between May 2019 and May 2024, and 35 negative controls were extracted and sequenced concurrently. Thirty-five food samples were excluded due to insufficient DNA concentration following extraction and library preparation or having less than one million reads post-read trimming and filtering (Fig. S1A), resulting in a total of 223 metagenomes for analysis (Table 1) alongside 35 negative controls. Samples purchased from butchers without package labelling, or for which the corresponding metadata were unavailable, were labelled as having an ‘unknown’ production method. Due to the similarities between some production methods, free-range and organic samples were grouped together as ‘extensive’ production and outdoor bred and conventional together as ‘intensive’. Wild-caught samples formed a third group, as samples that had not been farmed. Details of the 223 metagenomes included are shown in Table S1 (available in the online Supplementary Material).

**Table 1.** Sample metagenomes analysed within the project

Commodity	No. intensive samples (% commodity)	No. extensive samples (% commodity)	No. unknown samples (% commodity)	No. wild caught samples (% commodity)
Beef (*N*=33)	15 (45.5)	18 (54.5)	–	–
Lamb (*N*=33)	17 (51.5)	16 (48.5)	–	–
Pork (*N*=25)	7 (28.0)	12 (48.0)	6 (24.0)	–
Chicken (*N*=60)	22 (36.7)	26 (43.3)	12 (20.0)	–
Salmon (*N*=31)	14 (45.2)	5 (16.1)	–	12 (38.7)
Leafy greens (*N*=41)	25 (61.0)	15 (36.6)	1 (2.4)	–

Post read filtering, whole metagenome shotgun sequencing yielded an average of 324.5 million reads per sample (range: 1.34 million to 1.89 billion). Samples had on average ~2,491 times more bacterial reads identified compared to negative controls (after removing phiX reads, negative controls had 14,388 reads on average). Unclassified reads comprised 15.5% of reads on average across all samples. However, extensive salmon and intensive/extensive chicken were associated with a significant increase in the abundance of unclassified reads over intensive salmon (*P*=0.031, Mann–Whitney U test) and unknown chicken production (*P*=0.017 and <0.001, respectively) (Fig. S1B). Unclassified reads were removed from samples for downstream analyses.

### Composition of the microbiome

Across all samples, 26 phyla and 607 genera were identified. The predominant phyla varied between food commodities (Fig. S2). *Firmicutes* was the most abundant phylum identified on beef and lamb, comprising an average of 90.1 and 86.3% of classified reads, respectively, whereas *Pseudomonadota* (formerly *Proteobacteria*) was more abundant on chicken, leafy greens, pork and salmon at 72.3, 90.0, 77.8 and 85.3%, respectively.

The most abundant families on beef and lamb were *Streptococcaceae* forming 49.1 and 50.9% of the classified reads on average, respectively, and *Carnobacteriaceae* comprising 21.2 and 28.6%, respectively (Fig. S2). *Pseudomonadaceae* was the most abundant family identified on chicken, leafy greens, pork and salmon at 29.1, 52.7, 50.2 and 25.7%, respectively. On leafy greens, the second most abundant family identified was *Erwiniaceae* at 10.4%, while on chicken, pork and salmon, *Moraxellaceae* was more abundant at 26.5, 18.3 and 23.3, respectively.

**Fig. 1.**
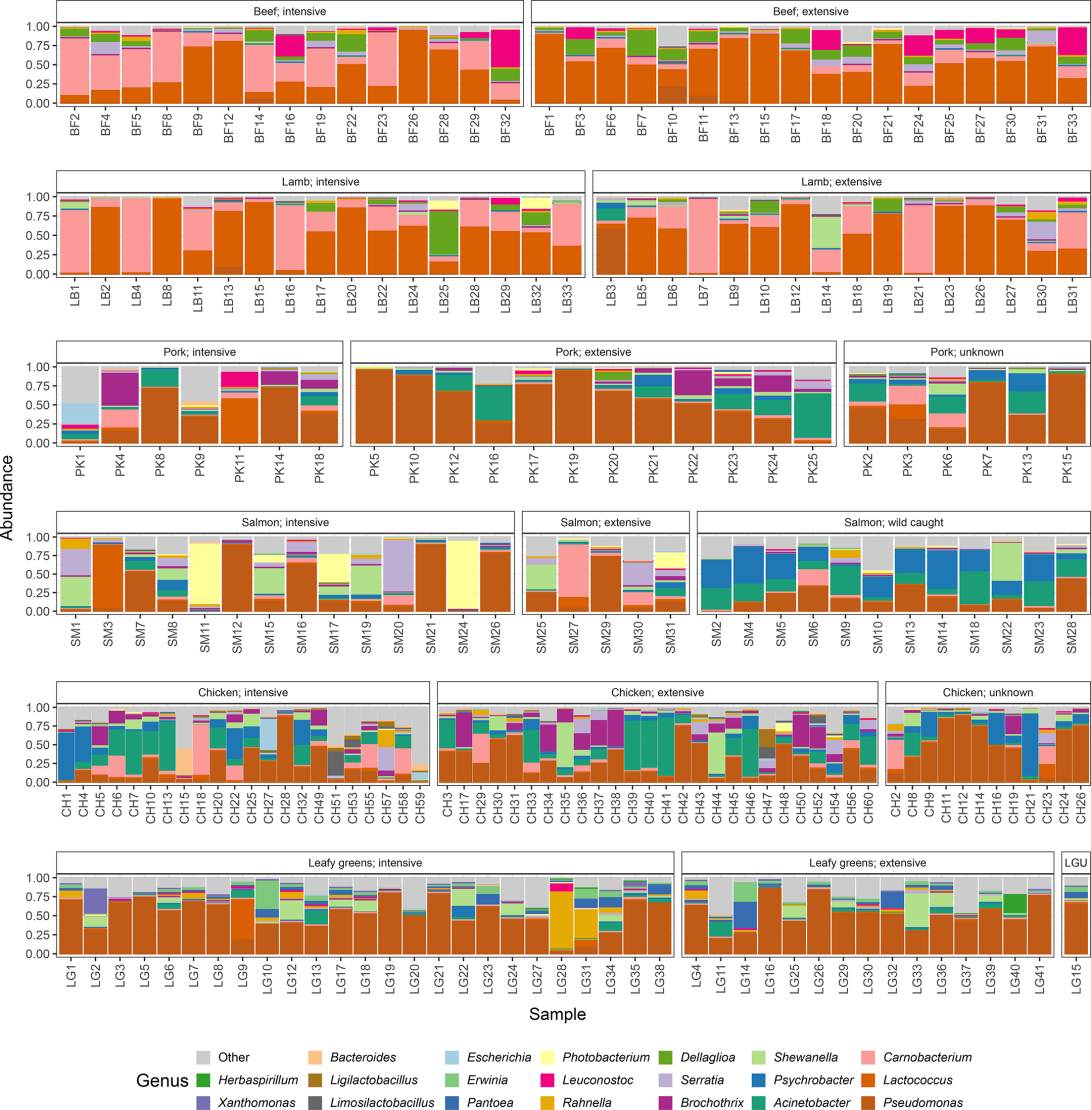
Proportions of bacterial genera identified from retail food product metagenomes. Unclassified reads were removed, and the remaining fraction was scaled to 1. Only the top 20 most abundant genera are shown here; less abundant organisms were grouped as ‘other’. LGU, leafy greens, unknown.

The most prevalent genus on beef and lamb was *Lactococcus*, comprising 49.1 and 50.9% of classified reads, respectively, followed by *Carnobacterium* at 21.2 and 28.6% and *Dellaglioa* at 10.4 and 5.43% (Fig. 1). On the remaining food commodities, *Pseudomonas* was the most common genus at 29.1% on chicken, 52.7% on leafy greens, 50.2% on pork and 25.7% on salmon. On chicken and pork, *Acinetobacter* was the next most common genus (16.4 and 13.4% respectively) followed by *Psychrobacter* and *Brochothrix* on each commodity, respectively (10.1 and 7.60%), while on leafy greens, *Shewanella* and *Pantoea* were more prevalent (6.13 and 5.63%). On salmon, *Psychrobacter* and *Acinetobacter* represented the next most common genus (13.1 and 9.97%).

A total of 22 genera associated with production methods were identified: 2 on beef, 4 on chicken and 16 on salmon (Fig. S3). Of these, 12 genera had an effect size greater than one, suggesting that the difference between groups was larger than the difference within groups. Associations between genera and production method on beef had median absolute effect sizes less than one (Table S2). *Leuconostoc* was more abundant on chicken samples with an unknown production method (average relative abundance=0.167%) compared to extensive chicken samples (average=0.002%; Benjamini-Hochberg corrected *P*-value=0.003). However, only two extensive chicken samples were positive for *Leuconostoc*.

Wild-caught salmon samples were enriched with *Weeksellaceae* over their farmed counterparts. *Kaistella* was more abundant in wild-caught salmon than in intensive and extensive samples (*P*=0.015 and 0.049, respectively), while abundance of *Algoriella* was significantly elevated compared to extensive samples (*P*=0.002). *Chryseobacterium* was also associated with wild-caught samples, but with an effect size less than 1 (Table S2). Wild-caught salmon also had a higher abundance of *Psychrobacter* over extensive salmon (*P*=0.015). Similarly, *Microbacterium* abundance was higher in wild-caught samples compared to both intensive and extensive varieties (*P*=0.004 and 0.042, respectively). Wild-caught salmon had higher abundance of *Pseudoclavibacter* (*P*=0.003)*, Stenotrophomonas* (*P*=0.049), *Leucobacter* (*P*=0.049) and *Brevundimonas* (*P*=0.049) over intensive, but lower abundance of *Aeromonas* (*P*=0.003) and *Serratia* (*P*=0.01). Finally, *Bacillus* abundance was elevated in extensive compared to intensive samples (*P*=0.049). However, no *Pseudoclavibacter*, *Brevundimonas* or *Bacillus* were identified in intensive samples.

Neither *Salmonella* nor *Listeria* was detected in any samples. *Campylobacter* was identified in 14 (23.3%) chicken samples, while *Vibrio* was detected in five (16.1%) salmon samples and two pork samples (Fig. S4). *Escherichia* and *Yersinia* were more prevalent, detectable in 23.8 and 75.8% of metagenomes, respectively. No associations were found between the presence/absence of these genera and production method (*P*>0.05 for all comparisons, Fisher’s Exact test).

### Microbial diversity

**Fig. 2.**
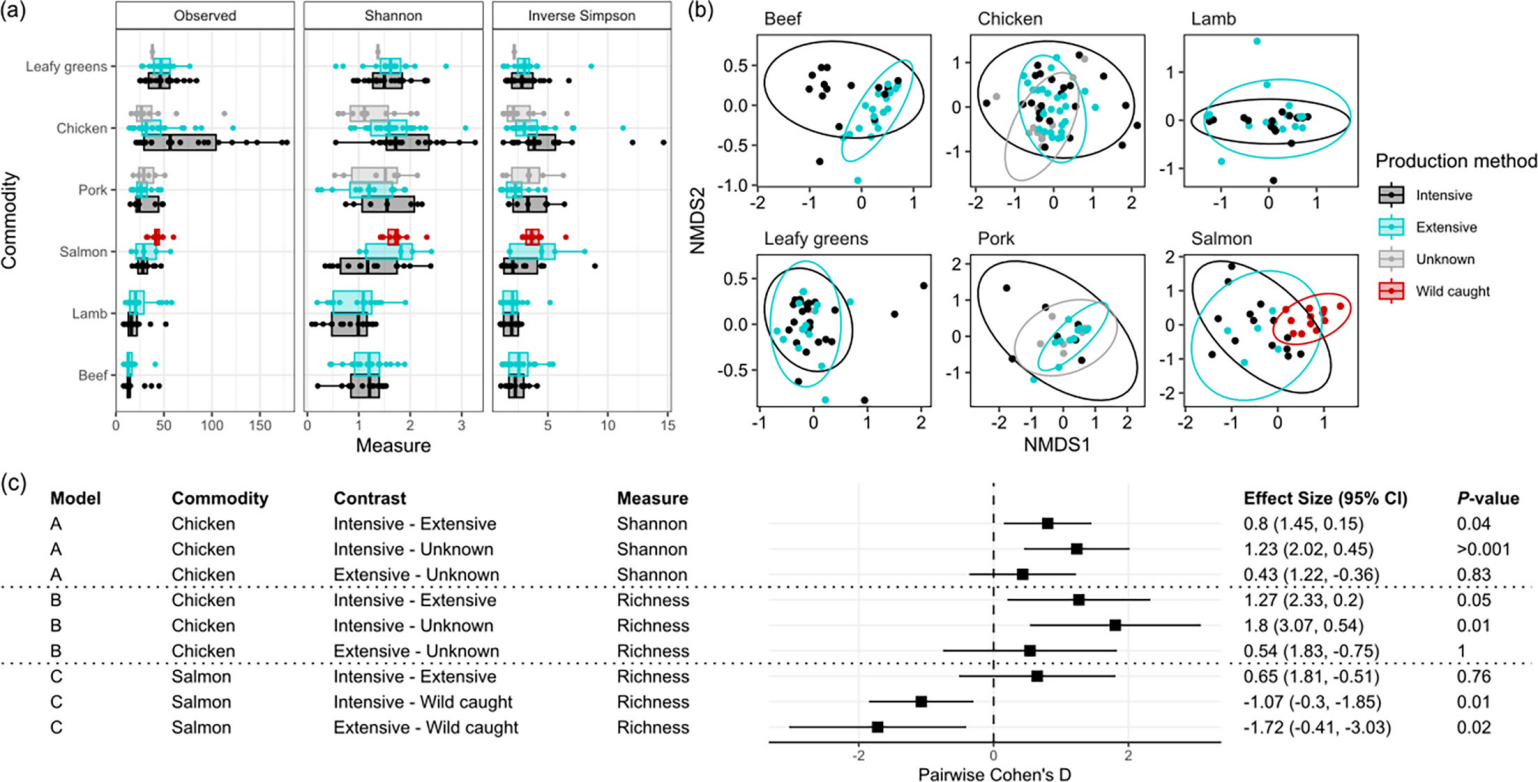
Taxonomic diversities and Bray–Curtis dissimilarities of retail food sample metagenomes, compared between production methods. (**a**) Diversity indices [species richness (observed), Shannon index, inverse Simpson’s index] of sample taxa agglomerated at the genus rank between production methods. (**b**) Bray−Curtis dissimilarities between genera were calculated and plotted using non-metric multidimensional scaling for each commodity. Samples are coloured by production method, and ellipses encompass samples from the same production method. Ordination stress: beef=0.11, lamb=0.09, chicken=0.20, leafy green=0.12, pork=0.12, salmon=0.18. (**c**) Forest plot of post hoc pairwise effect sizes (Cohen’s *D*) derived from LMs indicating a significant association between diversity indices and production method. Reference groups for each contrast are reported on the left of the comparison. Bonferroni correction was used to account for the effect of multiple pairwise comparisons on the *P*-values within each model. Production systems comprise intensive (conventional and outdoor-bred production), extensive (free-range and organic production), wild caught and unknowns.

Rarefaction curves suggested that the sequencing depth remained adequate to capture the taxonomic richness present on food (Fig. S5). LME models were used to assess the association of production method with microbial diversity measures for each commodity under study (Table S3). Samples from intensive and extensive beef, lamb, pork, leafy greens and salmon products shared similar Shannon index (Fig. 2a). However, on chicken, a significant association between microbial diversity (Shannon index) and production method (*P*=0.017) was identified post-correcting for multiple hypothesis testing. Pairwise analysis suggested intensively produced samples had a higher genus diversity than samples from unknown production methods (*P*=0.005) and extensive samples (*P*=0.041) (Fig. 2c). Similarly, the genus richness models suggested a significant association for chicken post-multiple correction (*P*=0.039), with a higher diversity of genera on intensively produced chicken compared to the unknowns (*P*=0.013) (Table S4).

Microbial richness was also associated with production method on salmon (*P*=0.028). Higher log richness was detected in wild caught compared to both intensive (*P*=0.013) and extensive (*P*=0.021) production methods (Fig. 2c). No associations were detected on other commodities (*P*>0.05, in all cases). Similarly, no associations were found between inverse Simpson’s index and production methods (*P*>0.05 in all cases) (Table S5).

To investigate between-sample differences in diversity, Bray–Curtis dissimilarities were calculated from relative abundances between samples from different commodities and production methods. Within each commodity, no significant difference was found between the centroids of each production method for lamb, leafy greens and pork (Fig. 2b). Significant differences were found between group centroids on beef (*P*=0.0024, PERMANOVA), chicken (*P*<0.001) and salmon (*P*<0.001), albeit with small effect sizes (difference between group centroids calculated as partial omega squared) ranging from 0.20 on beef to 0.066 on chicken (Table S6). Intensive beef had a higher dispersion [mean distance to centroid: 0.37, confidence interval (CI): 0.31, 0.43] than extensive (0.25, CI: 0.20, 0.30) (*P*=0.018, permutest), and wild-caught salmon had a lower dispersion (0.33, CI: 0.27, 0.40) compared to farmed varieties (intensive: 0.51, CI: 0.45, 0.57, extensive: 0.47, CI: 0.40, 0.53) (pairwise intensive *P*=0.001 and extensive *P*=0.017, permutest) (Fig. S6). Further analysis of clustering on chicken suggested significant separation between all three groups (*P*<0.05, in all cases, pairwise PERMANOVA).

### Composition of the resistome

**Fig. 3.**
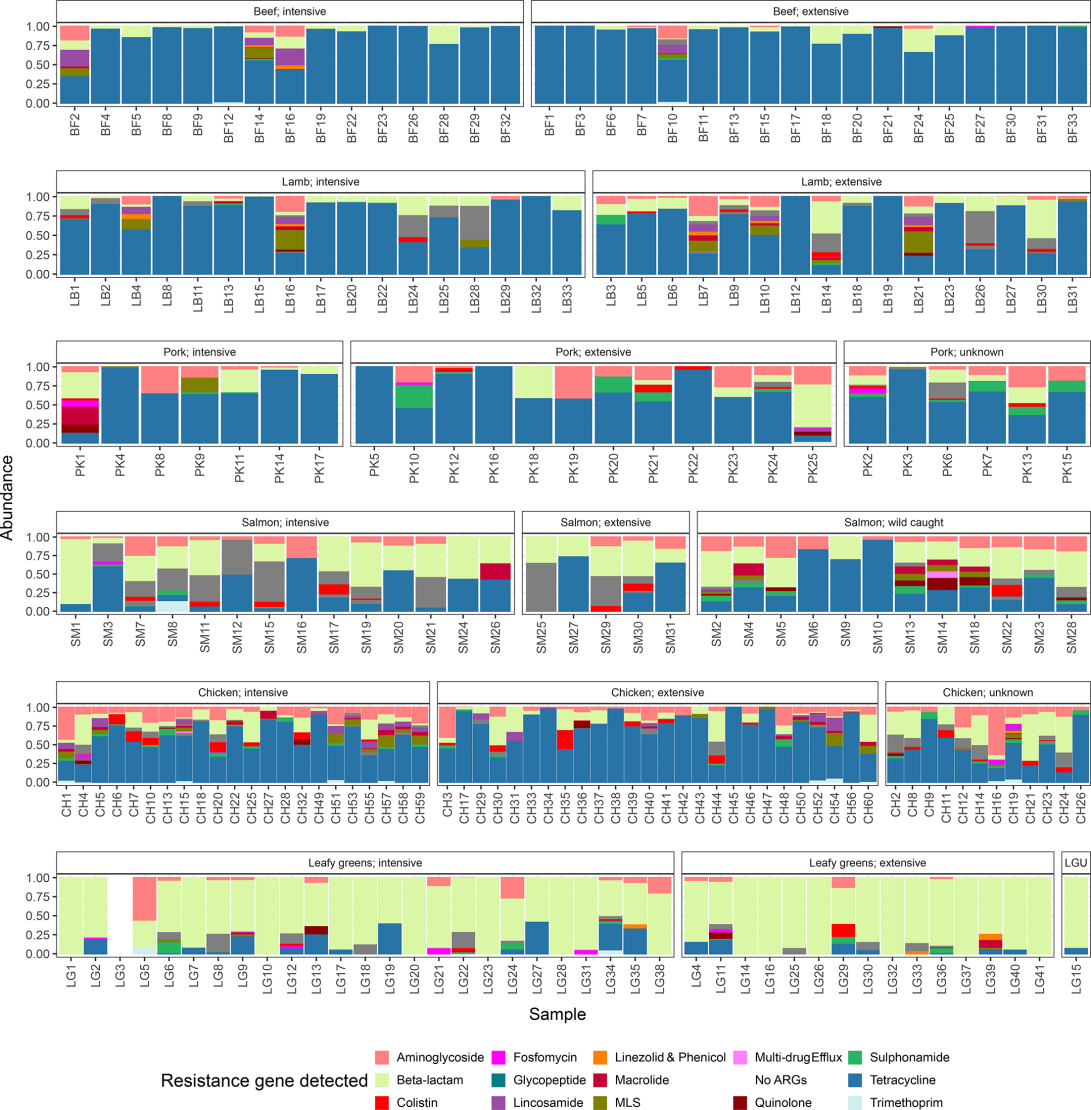
Antimicrobial class level composition of the resistome of retail food products. ARGs were identified using KMA against the ResFinder database, abundance was calculated as fragments per kilobase per million mapped reads and ARGs were grouped based on antimicrobial class towards which they conferred resistance. LGU, leafy greens, unknown; MLS, macrolides, lincosamides and streptogramin B.

A total of 284 unique ARGs were identified putatively conferring resistance to 18 antimicrobial drug classes. One sample had no detectable ARGs (leafy greens from intensive production). Beef production was predominated by tetracycline resistance, representing 91.9% of the resistome on average (Fig. 3). Tetracycline resistance was less predominant on lamb, chicken and pork, although it still made up a majority of the resistome at 65.7% in lamb, 61.0% in chicken and 71.2% in pork. Leafy green and salmon samples possessed a more distinct ARG community composition compared to meat samples. On leafy greens, genes conferring beta-lactam resistance comprised 79.9% of the resistome on average, while on salmon, beta-lactam and tetracycline resistance comprised 56.5 and 20.7% of the resistome, respectively. However, this was highly variable between samples (Fig. 3). Excluding beef, all other commodities possessed a detectable abundance of colistin resistance genes (Fig. S7). This was highest in chicken and salmon forming 2.31 and 3.72% of the total resistome, respectively.

No significant associations were found between the relative abundance of individual ARGs and production method (*P*>0.05 in all cases, ALDEx2). ARG composition was highly variable between samples (Fig. S7).

### Resistome diversity

**Fig. 4.**
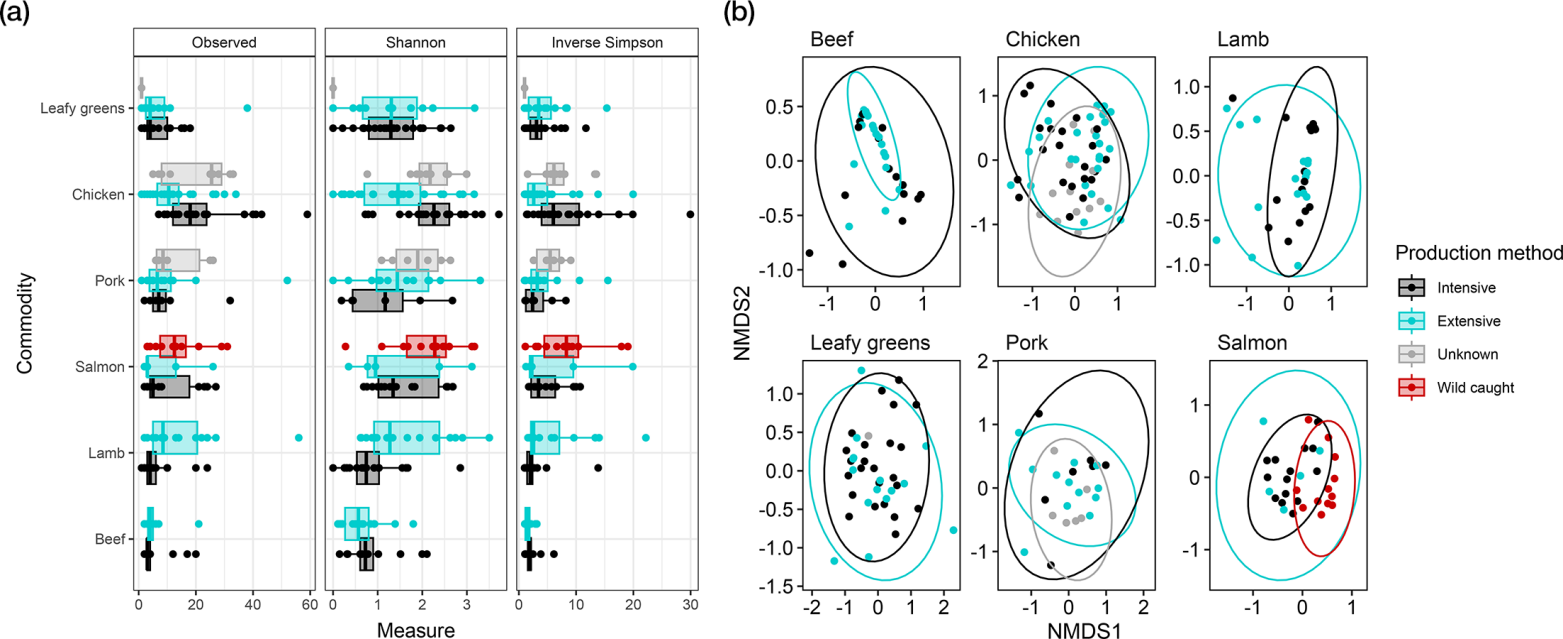
ARG diversities and Bray–Curtis dissimilarities between retail food metagenomes from different production systems. (**a**) Diversity indices [species richness (observed), Shannon index, inverse Simpson’s index] of sample ARGs between production methods and commodities. (**b**) Bray–Curtis dissimilarities between sample ARGs plotted using non-metric multidimensional scaling for each commodity. Samples are coloured by production method, and ellipses encompass samples from the same production method. Ordination stress: beef=0.06, lamb=0.13, chicken=0.18, leafy greens=0.15, pork=0.18, salmon=0.17. Production systems comprise intensive (conventional and outdoor-bred production), extensive (free-range and organic production), wild caught and unknowns.

No associations between ARG alpha diversity measures and production methods were identified (*P*>0.05 in all cases) (Tables S7, S8 and S9). However, rarefaction curves suggested the theoretical maximum number of unique ARGs was not observed for any commodity investigated and that greater sequencing depth may have yielded greater ARG richness overall (Fig. S8). For beta diversity, statistical analysis suggested a significant association between production method and Bray–Curtis dissimilarity on chicken (*P*=0.0048, Adonis2) and salmon (*P*=0.0066) (Fig. 4b), with effect sizes of 0.054 and 0.080, respectively (Table S10). Pairwise analysis between production methods suggested that chicken samples from extensive production systems were distinct from samples from intensive systems (*P*=0.032, pairwise Adonis2) and unknowns (*P*=0.0093). Similarly, wild-caught salmon samples clustered separately from farmed varieties (intensive *P*<0.001, extensive *P*=0.039). Samples from other production methods overlapped and did not form unique clusters post-multiple correction (*P*>0.05 in all cases, Adonis2). Differences in the dispersion of samples around group centroids were identified between production systems on beef (*P*=0.006, permutest). No significant differences were identified between group dispersions for other commodities (*P*>0.05 in all cases, permutest) (Fig. S9).

The ARGs detected here may be encoded chromosomally or on mobile genetic elements, an example of which is plasmids. A positive association was found between the number of unique plasmid replicons identified on rarefied chicken samples and production method (*P*=0.045) (Fig. S10). Intensive chicken samples had a higher number of unique replicons compared to extensive (*P*=0.006) with a mean difference of 0.89 (CI: 0.22, 1.56). No associations were detected between intensive/extensive and unknown production (*P*>0.05 in both cases). No associations were detected on other commodities between production method and the number of unique plasmid replicons (Table S11).

### Concentration of ARGs and bacteria on foods

**Fig. 5.**
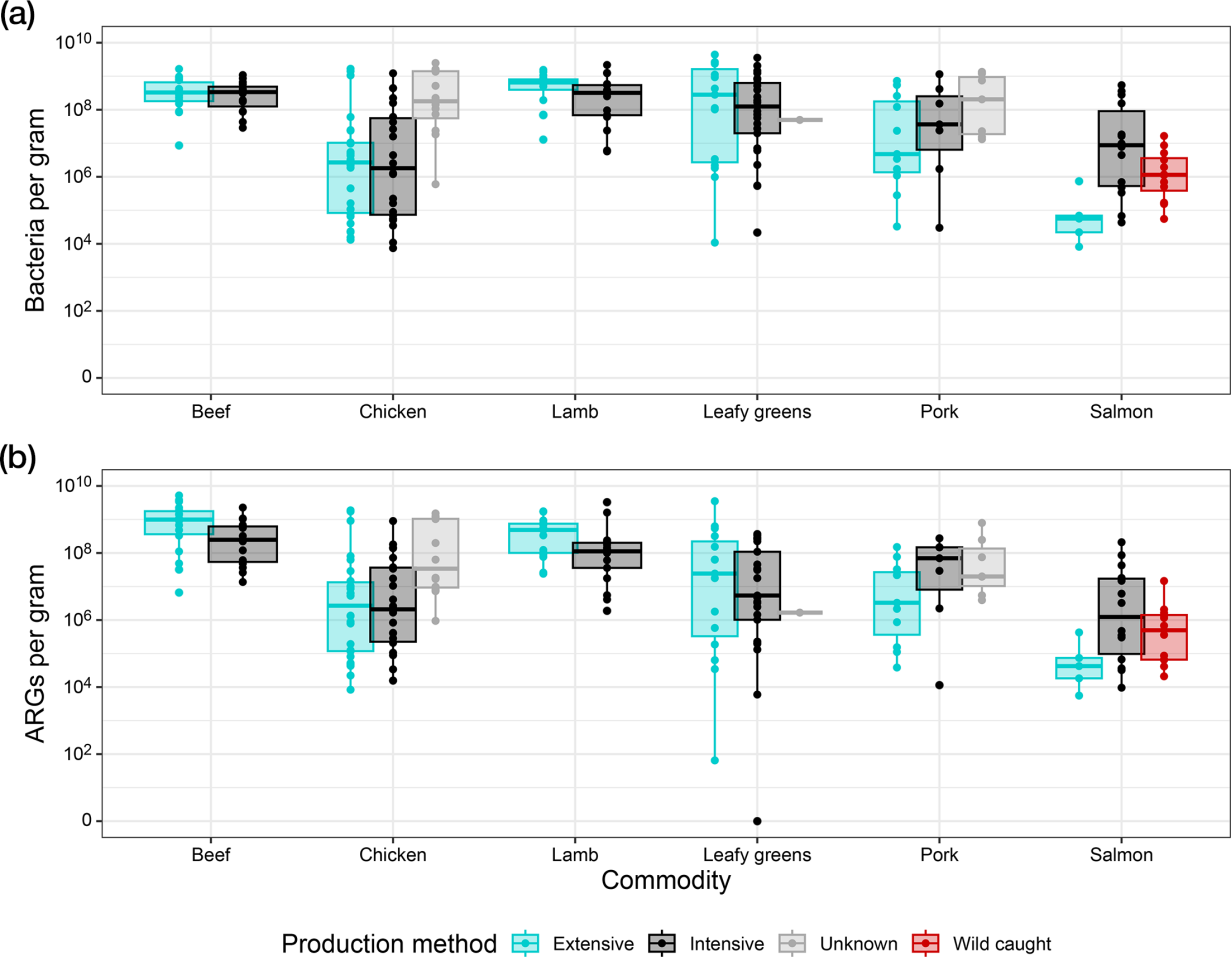
Log 10 transformed bacterial (**a**) and ARG (**b**) concentrations per gram of retail foods. Production systems comprise intensive (conventional and outdoor-bred production), extensive (free-range and organic production), wild caught and unknowns.

The concentration of bacteria varied between production methods (Fig. 5a). The distribution of ARG concentrations between production methods was similar to the bacterial concentrations (Fig. 5b). However, post-multiple correction, no significant associations were identified between ARG or bacterial concentrations and production methods (*P*>0.05 in all cases, LME; Tables S12 and S13).

## Discussion

We investigated differences in the microbiome and resistome of intensively and extensively produced retail products collected in Norfolk, UK, across a range of food commodities. For a majority of food commodities, we did not find evidence for differences in the burden of AMR as measured by ARGs on retail foods between different farming practices. Food samples from different production systems possessed a similar microbiota, although 22 genera associated with production method on beef, chicken and salmon were detected.

Intensively produced chicken had more diverse microbiomes by Shannon index and richness compared to extensive and unknown samples. This likely derives from differences in how these measures are calculated; each method differently weights the effect of evenness (relative abundance of genera) on the result, such that the effect of rare taxa is highest for richness and lowest for inverse Simpson’s index. The observed associations may therefore derive from increased numbers of rare taxa in intensive chicken compared to other varieties or may be an artefact of this set of samples. Differences in bacterial diversity on chicken were coupled with differences in the clustering of samples between production methods using taxonomic Bray–Curtis dissimilarities. Similar clustering of samples by production method was observed on salmon and beef, although this was coupled with associations between dispersion distances and production method. This suggests that the clustering observed on salmon and beef may stem from differences in dispersion, and not a true distinction between production methods. However, the increased proportion of unclassified reads from extensive salmon over other production methods may represent uncharacterised microbial diversity contaminating differentially produced salmon at retail.

Analysis of microbial abundance between production methods using ALDEx2 identified 22 genera potentially associated with production methods. However, except for associations with *Kaistella*, all CIs calculated by ALDEx2 included zero (Table S2), suggesting no significant difference. The *P*-values for these associations are reported in the main text due to inconsistencies in how the confidence intervals and the point estimates were calculated. Disparity between confidence intervals and *P*-values suggests that the results may not be conclusive, and as such, more data are needed to properly characterise these effects.

On wild-caught salmon, 16 genera with significantly different relative abundance compared to aquacultured varieties were identified, and wild-caught samples had a higher richness and lower dispersion of genera compared to their farmed counterparts. These differences may suggest that captive-raised salmon possess a distinct microbiota over fish from wild habitats, as has been previously suggested [48,49]. However, wild-caught salmon products were labelled as different species (Pacific salmon, *Oncorhynchus* spp.) than their farmed counterparts (Atlantic salmon, *Salmo salar*). Previous research has identified differences in gut microbiomes of *S. salar* and *Oncorhynchus mykiss* (rainbow trout), suggesting that the different host species may have contributed to the observed differences within this study [50]. Alternatively, most wild-caught samples were previously frozen (11 out of 12 compared to 2 out of 29 farmed samples), which may select for a narrower range of psychrophilic organisms compared to other storage methods. From our data alone, it is unclear whether or not these differences in bacterial contamination constitute a food safety risk.

Of the genera identified as differentially abundant between production methods, *Aeromonas*, *Acinetobacter* and *Serratia* are known to include opportunistic pathogens [51,53]. From this study, it is unclear whether or not differences in the abundance of these organisms may present a threat to public health. Confidently identifying foodborne pathogens in short-read metagenomes has previously proven difficult due to the generally low abundance of these organisms [18,54]. Therefore, it is likely that samples contaminated with pathogens below the level of detection will not have been identified; if that is the primary aim, microbial culture is a more sensitive method for detecting pathogens contaminating retail foods [55]. Additionally, differences in the abundance of genera containing spoilage-associated organisms were identified, such as *Leuconostoc* and *Psychrobacter*, suggesting farming and other production practices may influence the shelf life of some food commodities. However, given the large confidence intervals and observational nature of the study, we cannot be certain that our findings reflect real effects of production method on bacterial abundances.

Our analysis did not detect differences in ARG alpha diversity between production methods for any of the commodities investigated, in line with previous findings on chicken [17]. Metagenomic profiling of ARG diversity has been demonstrated to be heavily dependent on sequencing depth [56]. While deeper sequencing of food metagenomes may have yielded a greater overall ARG richness, we accounted for variations in sequencing depth between samples by including sequencing depth as a covariate in the alpha diversity modelling approach. Distinct clustering of ARG Bray–Curtis dissimilarities was detected between different production systems on chicken and salmon. This suggests that consumers of intensively produced chicken or wild-caught salmon may be exposed to potentially different populations of ARGs. However, it is unclear whether or not this constitutes an increase in risk, given no associations were found between production method and ARG concentration nor the relative abundance of individual ARGs (using ALDEx2). Furthermore, the food samples tested in this study were raw and intended to be cooked (excluding leafy greens samples). There is limited evidence on the impact of cooking on ARGs available for transfer to other microbes in the human gut after consumption [57].

No associations were detected between production method and ARG relative abundance, nor concentration, suggesting that production method may not influence the ARG composition of the retail food products investigated here. While the most abundant ARGs detected in our study conferred putative resistance towards the most commonly used antimicrobials in livestock farming, namely, tetracyclines and beta-lactams [58], these are amongst the most commonly detected ARGs across the One Health spectrum [16,59, 60]. Given previous evidence indicates the food processing environment is important for shaping the food microbiome and resistome, these processing effects may be more impactful than differences in farming practices derived from production systems [14,16, 37]. A high degree of variation was observed between samples of the same production method, which may stem from the influence of other factors, such as retail outlet, or supplier, on the food microbiota and/or resistome. Such variation made discerning between noise and true differences between production methods difficult, highlighting the complexity of food production and the need to consider potentially confounding factors within analyses.

Our study is subject to a number of potential limitations. The production method was not known for some samples, particularly those collected from butchers’ shops (Table S1). Previous research has suggested links between the food microbiome and the type/size of store selling it [61]. Here, the bacterial concentration of samples from unknown production systems was elevated over extensive and intensive samples, while the taxonomic alpha diversity was lower. These differences may stem from the store type, although this relationship could not be tested using the data collected here because of the low number of samples collected from butchers. Due to the small sample sizes for some groups of pork and salmon, low statistical power and low effect sizes prevented robust conclusions from being drawn, and more data are required to characterise the relationship fully between production methods for these commodities. As this investigation was conducted on retail foods, the sources of the bacteria or ARGs detected here cannot be ascertained along the food processing chain. Similarly, in many cases, the effect of factors acting further up the processing chain cannot be accounted for. Previous research found distinct microbial profiles on chicken from different processing facilities, suggesting the supplier may be an important driver of microbial variation on foods [19]. Furthermore, some processing facilities may specialise in either conventional or organic products; information was not available for which samples may have derived from such specialised suppliers.

It is important to note that all samples were collected from a single region (Norfolk, UK), although the majority of these were collected from large retail chains and thus are likely to be representative of those available in the rest of the country. In the UK, overall sales of antimicrobials for use in food producing animals have dropped 57% since 2014, with a decrease of 84% in sales of highest priority critically important antimicrobials over the same time period [58]. Sampling from other countries, where there may be different regulations governing organic food production and antimicrobial stewardship within agriculture, may yield differing results.

## Conclusions

Taken together, our results suggest that by the end of the food processing chain, production method was not a major driver of ARG abundance nor diversity across the majority of the commodities investigated here. This suggests other factors may be more important in shaping the retail food resistome. While there was little difference in AMR, more research investigating the differentially abundant microbes identified here is needed to understand whether their abundance on food is associated with differential clinical risks or spoilage effects. Given the increasingly globalised food supply, utilising a similar framework in other countries will extend our understanding of the potential impacts of different food production systems on AMR in foods.

## Supplementary material

10.1099/mgen.0.001705 Uncited Supplementary Material 1.

10.1099/mgen.0.001705 Uncited Supplementary Material 2.

## Abbreviations

AMR: antimicrobial resistance
ARG: antimicrobial resistance gene
BPW: buffered peptone water
CI: confidence interval
LM: linear model
LME: linear mixed effects model
PERMANOVA: permutational multivariate ANOVA
qPCR: quantitative PCR
