# Supplementary material for: Metagenomic analysis of UK retail foods finds limited evidence for associations between food production method and antimicrobial resistance gene burden
Source: Microb Genom. 2026 Apr 29;12(4):001705. doi: 10.1099/mgen.0.001705 (PMC13135472; doi:10.1099/mgen.0.001705)
Supplement: Uncited Supplementary Material 1. [file mgen-12-01705-s001.pdf]

Metagenomic analysis of UK retail foods finds limited evidence for associations between food production method and antimicrobial resistance gene burden – supplementary figures

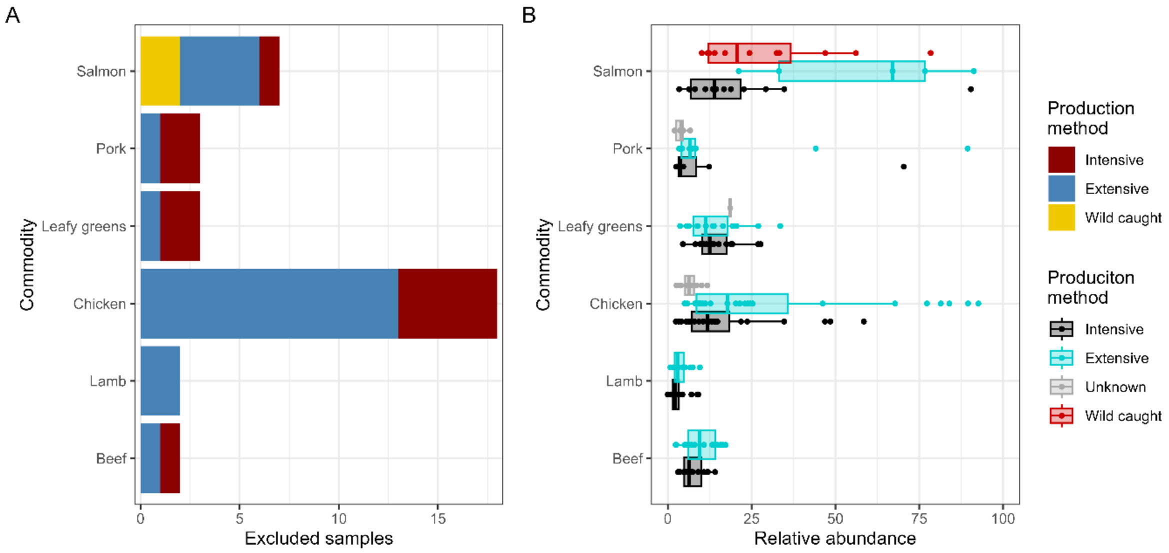

Figure S1: Distribution of excluded samples and unclassified reads. **A:** Number of samples excluded from analysis due to either low DNA concentrations, or low numbers of reads post sequencing. **B:** Relative abundance of unclassified reads per sample between production methods for each commodity. Production systems comprise intensive (conventional and outdoor-bred production), extensive (free-range and organic production), wild caught, and unknowns.

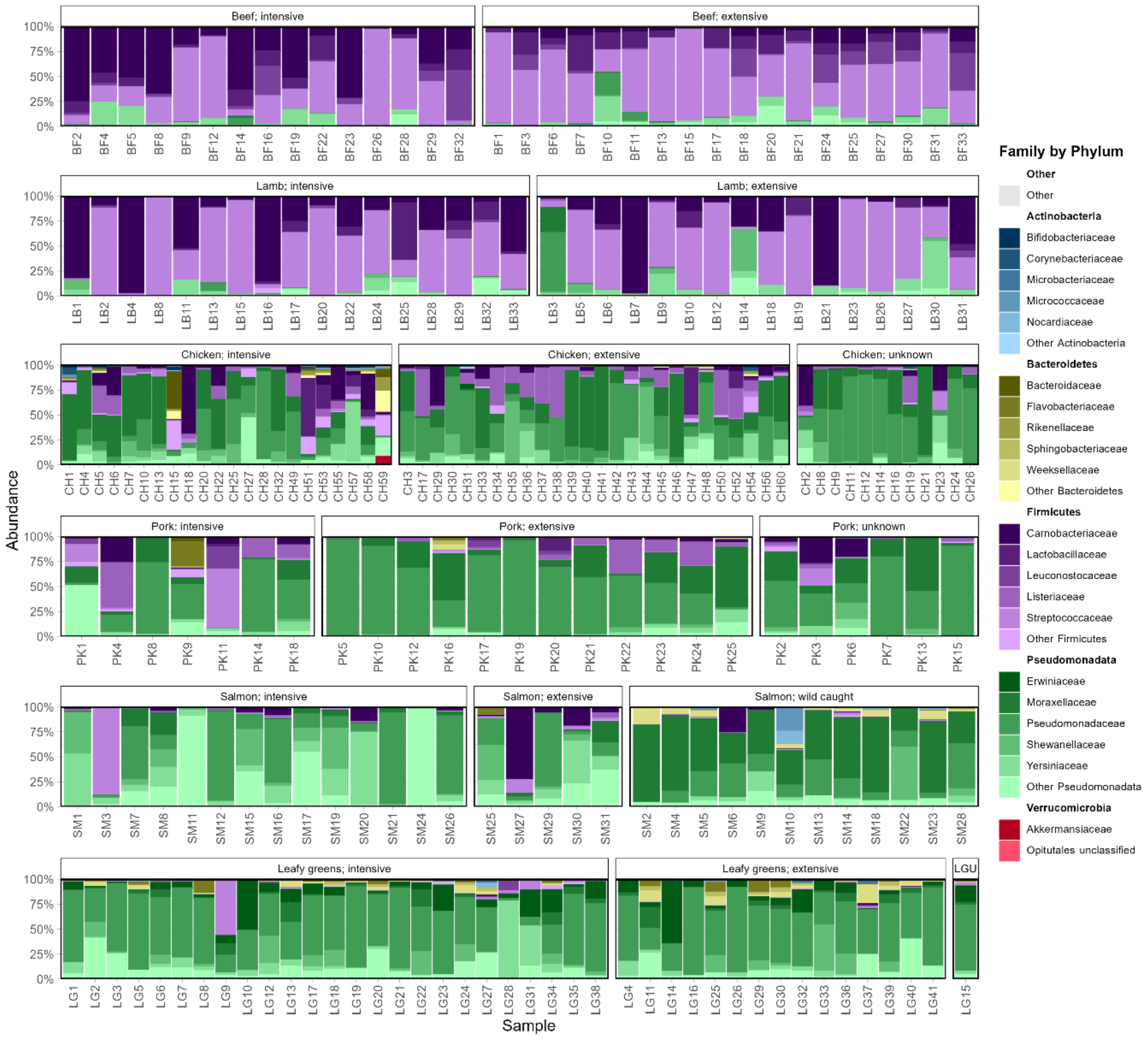

Figure S2: Relative abundance of families and phyla identified within food metagenomes. Bars represent relative abundance of bacteria from each sample post filtering and are coloured by the top 5 phyla, with the top 5 families of each nested within. 'Other' indicates reads classified as bacteria that were not assigned to a phylum. LGU = Leafy greens; unknown.

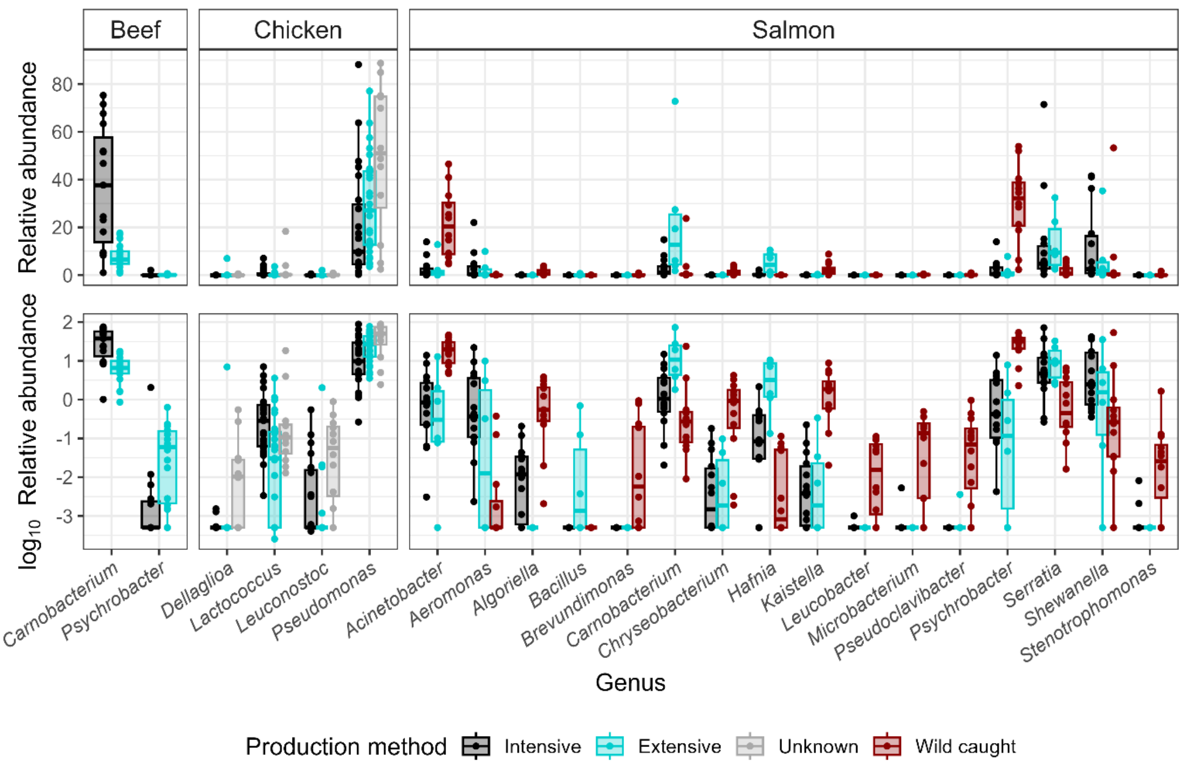

Figure S3: Significant differential abundance analysis results from ALDEx2. Boxplots represent the (raw and log_{10} transformed) relative abundance of classified reads for Benjamini-Hochberg-corrected significant hits identified by ALDEx2. Only samples with greater than zero abundance for each taxon are shown for log_{10} transformed results.

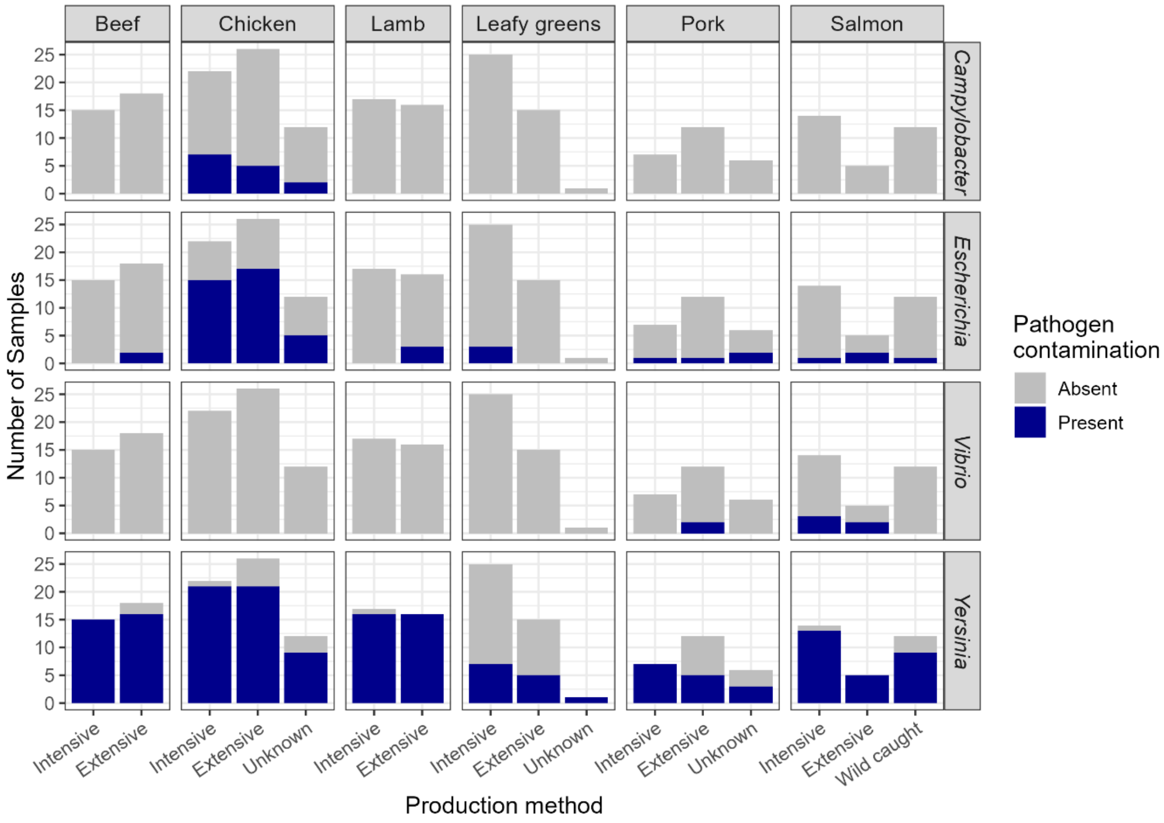

Figure S4: Number of samples positive for foodborne pathogens. Bars represent the total number of samples from each commodity and production method of coloured by the presence of genera containing foodborne pathogens (*Listeria, Campylobacter, Salmonella, Escherichia, Vibrio, Yersinia*). Neither *Listeria*, nor *Salmonella* were detected in any samples.

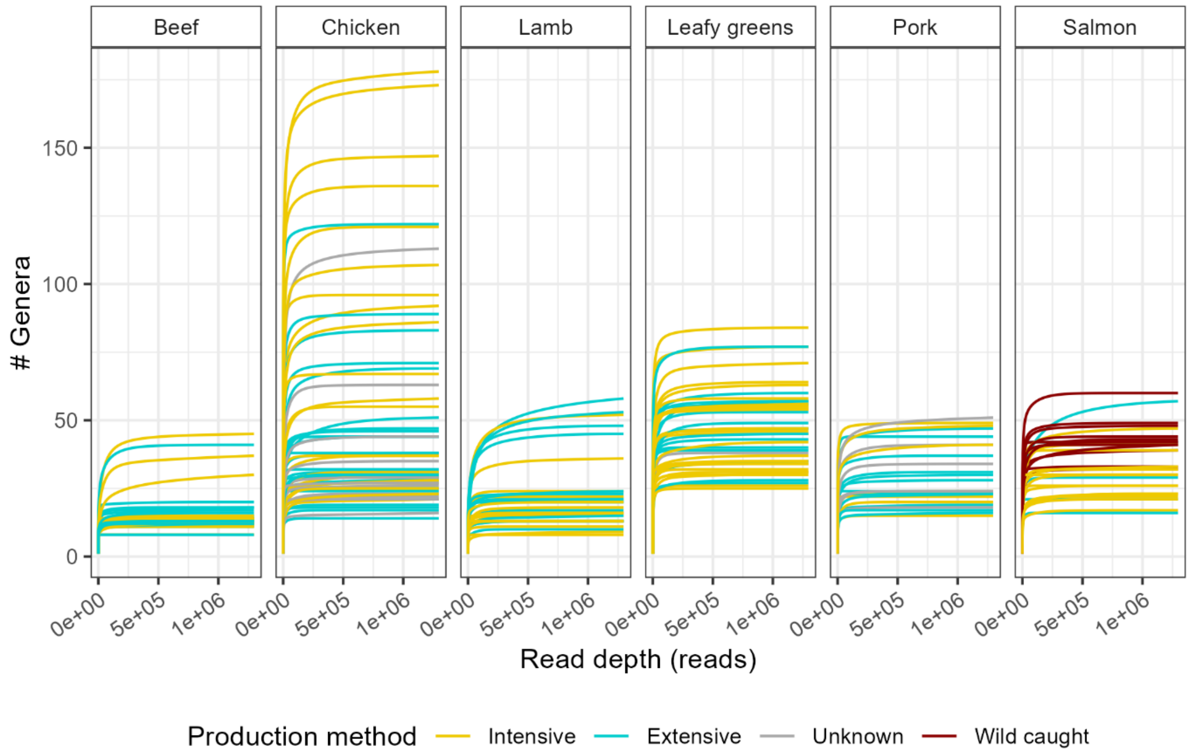

Figure S5: Rarefaction curves of microbial taxonomic data. Samples with less than one million reads were removed, and reads were rarefied to the depth of the smallest sample. Lines are coloured by production system, which comprises intensive (conventional and outdoor-bred production), extensive (free-range and organic production), wild caught, and unknowns.

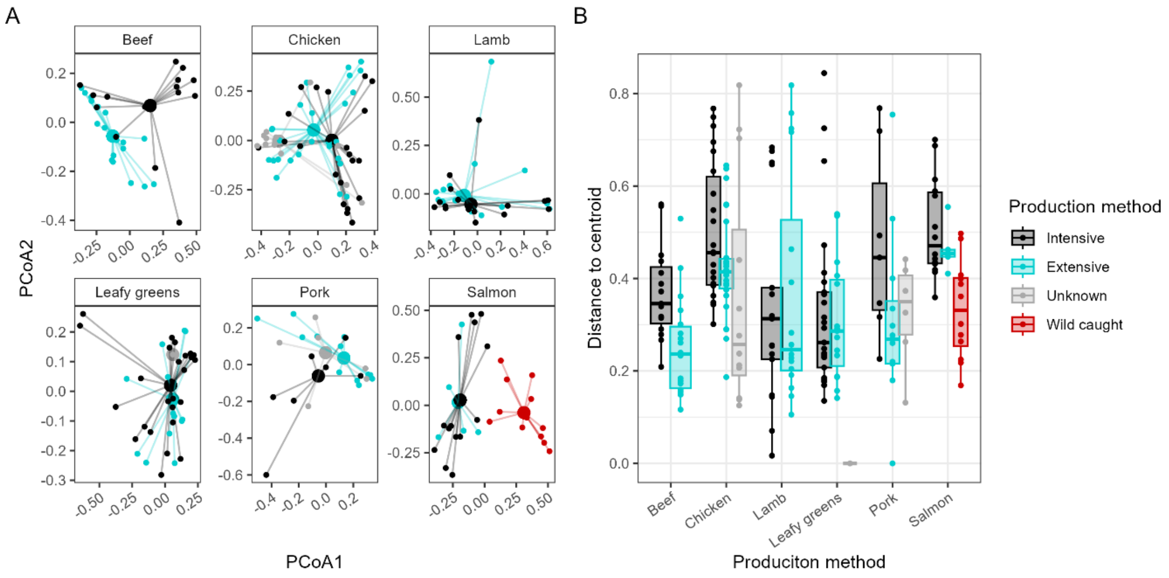

Figure S6: Dispersion between production methods from Bray-Curtis distances calculated between sample genera. **A:** Principal coordinate analysis (PCoA) of the dispersion of samples around each group (production method) centroid. Centroids are represented by a central larger point, with lines connecting to samples of the same production method **B: D**istances between each sample and its corresponding centroid from the PCoA.

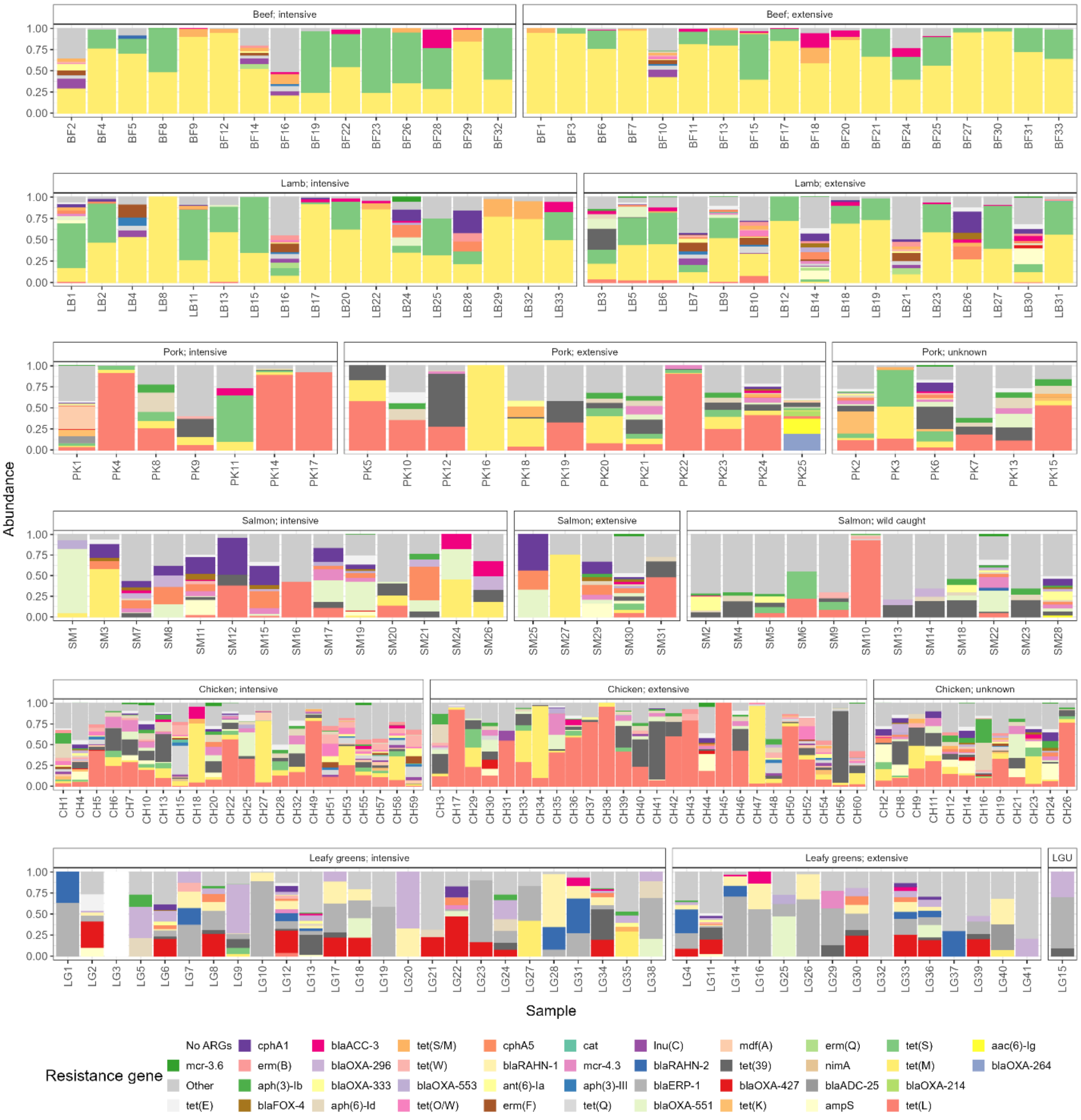

Figure S7: Relative abundance of antimicrobial resistance genes (ARGs) identified within food metagenomes. Bars represent the composition of the top 20 most abundant ARGs identified in food metagenomes. Less abundant ARGs were grouped as ’Other’.

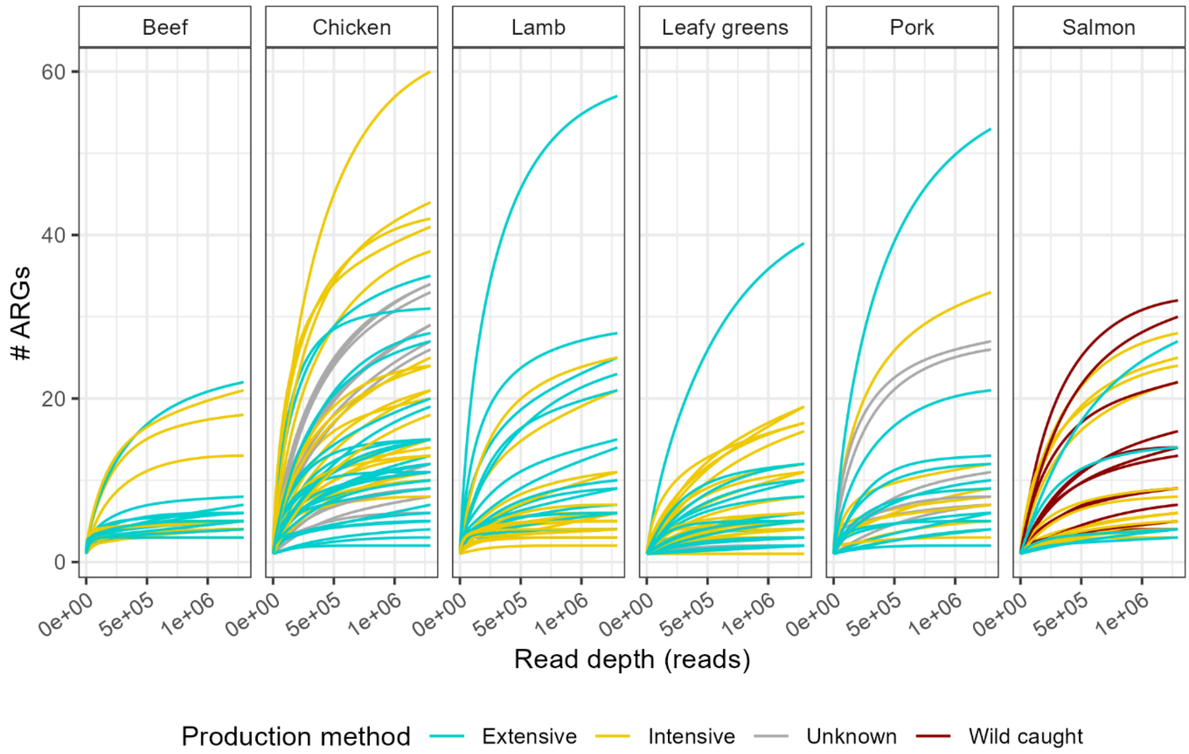

Figure S8: Rarefaction curves of antimicrobial resistance gene data. Samples with less than one million reads were removed and reads were rarefied to the depth of the smallest sample. Lines are coloured by production system, which comprises intensive (conventional and outdoor-bred production), extensive (free-range and organic production), wild caught, and unknowns.

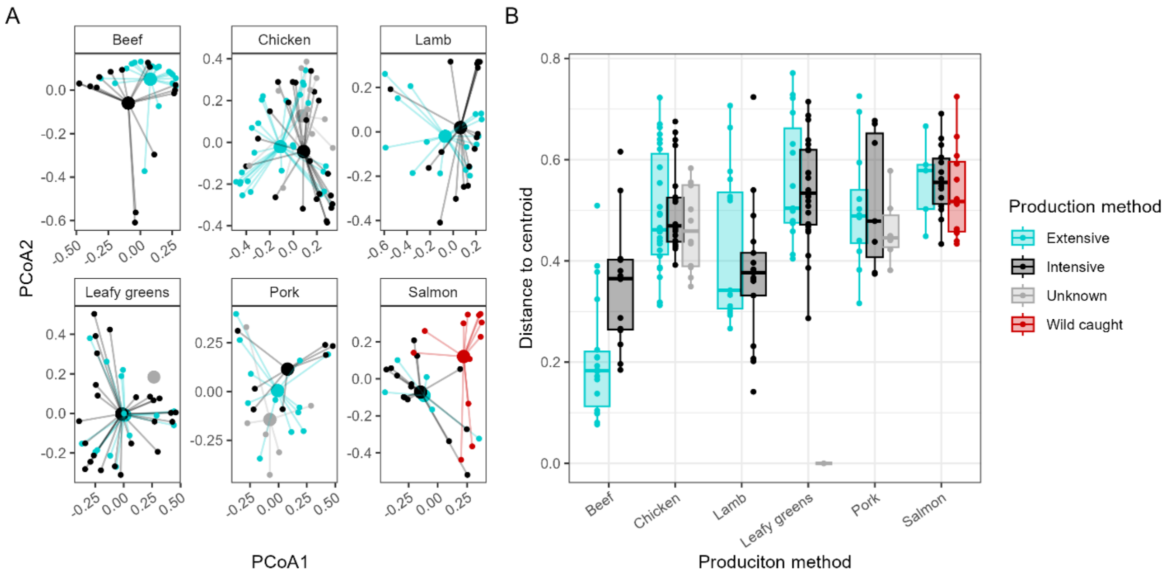

Figure S9: Dispersion between production methods from Bray-Curtis distances calculated between sample antimicrobial resistance genes (ARGs). **A:** Principal coordinate analysis (PCoA) of the dispersion of samples around each group (production method) centroid. **B:** Distances between each sample and its corresponding centroid from the PCoA.

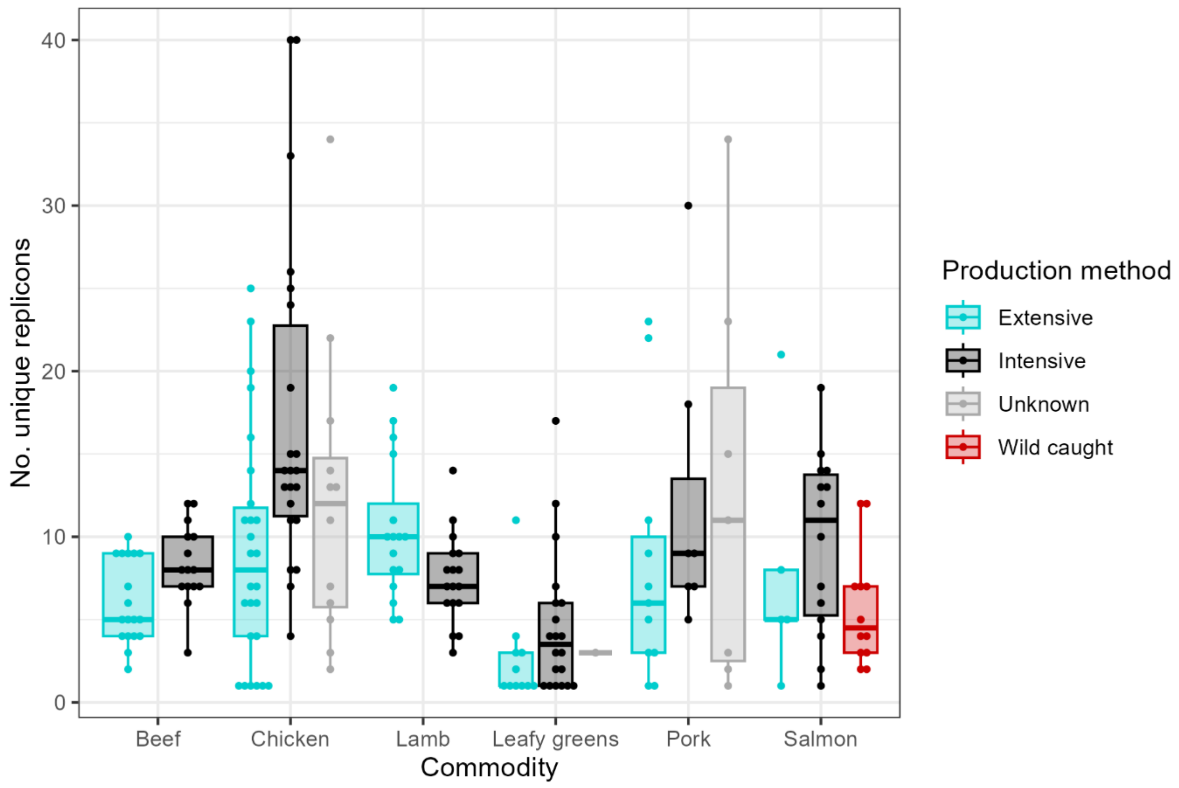

Figure S10: Number of unique plasmid replicons identified per sample between production systems for each commodity.
